# Respiratory Syncytial Virus Assembles into Structured Filamentous Virion Particles Independently of Host Cytoskeleton and Related Proteins

**DOI:** 10.1371/journal.pone.0040826

**Published:** 2012-07-13

**Authors:** Fyza Y. Shaikh, Thomas J. Utley, Ryan E. Craven, Meredith C. Rogers, Lynne A. Lapierre, James R. Goldenring, James E. Crowe

**Affiliations:** 1 Department of Pathology, Microbiology and Immunology, Vanderbilt University Medical Center, Nashville, Tennessee, United States of America; 2 Department of Pediatrics, Vanderbilt University Medical Center, Nashville, Tennessee, United States of America; 3 Department of Surgery and the Epithelial Biology Center, Vanderbilt University Medical Center, Nashville, Tennessee, United States of America; 4 The Medical Scientist Training Program, Vanderbilt University Medical Center, Nashville, Tennessee, United States of America; 5 The Vanderbilt Vaccine Center, Vanderbilt University Medical Center, Nashville, Tennessee, United States of America; University of North Carolina at Chapel Hill, United States of America

## Abstract

Respiratory syncytial virus (RSV) is a single-stranded RNA virus that assembles into viral filaments at the cell surface. Virus assembly often depends on the ability of a virus to use host proteins to accomplish viral tasks. Since the fusion protein cytoplasmic tail (FCT) is critical for viral filamentous assembly, we hypothesized that host proteins important for viral assembly may be recruited by the FCT. Using a yeast two-hybrid screen, we found that filamin A interacted with FCT, and mammalian cell experiments showed it localized to viral filaments but did not affect viral replication. Furthermore, we found that a number of actin-associated proteins also were excluded from viral filaments. Actin or tubulin cytoskeletal rearrangement was not necessary for F trafficking to the cell surface or for viral assembly into filaments, but was necessary for optimal viral replication and may be important for anchoring viral filaments. These findings suggest that RSV assembly into filaments occurs independently of actin polymerization and that viral proteins are the principal drivers for the mechanical tasks involved with formation of complex, structured RSV filaments at the host cell plasma membrane.

## Introduction

Respiratory syncytial virus (RSV) is a leading cause of serious viral lower respiratory tract illness in infants and the elderly worldwide. The virus is a member of the *Paramyxoviridae* family, and the genome consists of a single-stranded, negative-sense RNA molecule that encodes 11 proteins. The genome encodes three glycoproteins, six internal proteins, and two non-structural proteins. The fusion (F) protein is sufficient for mediating viral entry into cells *in vitro*. After viral and cell membrane fusion occurs, the viral polymerase complex, consisting of the nucleoprotein (N), the phosphoprotein (P), and the large (L) polymerase protein, and matrix protein 2, alternate reading frame 1 (M2-1), transcribes viral mRNA from the negative-strand RNA genome [1]. Glycoproteins are translated and trafficked through the secretory pathway to the apical surface, while internal virion proteins are translated in the cytoplasm [2], [3]. Generation of nascent RSV genomic RNA appears to occur in discrete cytoplasmic inclusion bodies that contain the matrix (M) protein and the viral polymerase proteins [4], [5]. It is suspected that N and P form ribonucleoprotein (RNP) complexes with the genomic RNA in inclusion bodies, possibly with the M protein, then traffic to the apical cell surface where they meet the glycoproteins. At the cell surface, viral proteins assemble into viral filaments that contain both viral structural proteins and viral genomic RNA [6]–[8]. These filaments are thought to assemble into infectious virion particles prior to separation from the cell surface, since they can contribute to cell-cell spread of the virus and morphologically resemble the filamentous form of virions seen in electron micrograph studies of virus produced in polarized cells [7]. Viral budding occurs in a Vps-4 independent manner [7] resulting in pleomorphic particles ranging from 150–250 nm in diameter for spherical forms and up to 10 µm long in filamentous forms [9].

Although many aspects of the viral life cycle have been studied, the mechanisms by which RSV assembles and buds are not well defined. For viral assembly, the viral structural proteins and the viral RNA must traffic to the cell surface and coordinate with viral glycoproteins in order to assemble into filaments and form free virions. It has been shown previously that RSV matures at the apical surface of infected cells [6]. RSV F protein can traffic to the apical surface independent of other viral proteins as directed by the transmembrane domain [10], and mutations in the cytoplasmic domain of F affect viral assembly into filaments [8]. RSV N localizes underneath the apical surface independent of viral glycoproteins [11]. It was suggested recently that the M protein may be responsible for targeting the viral RNP complex to the cell surface from inclusion bodies, which likely are the sites of RNA replication [12].

In addition to viral determinants, many host proteins have been implicated in the process of virus assembly and budding. Disruption of the function of apical recycling endosome (ARE) proteins myosin Vb and Rab11-family interacting protein 1 (FIP1) results in decreased production of viral progeny [3]. Additionally, inhibition of Rab11-FIP2 caused an increase in cell-associated infectious virus and longer viral filaments on cells, suggesting a defect in membrane scission to produce free virions [7]. Lipids and lipid-associated proteins have been implicated to play a role in facilitating RSV assembly [1], [13]. RSV proteins associate with detergent resistant membranes, and RSV proteins co-localize with lipid microdomain markers [2], [3], [13]–[15]. It is therefore presumed that RSV filaments form at lipid microdomains.

Since lipid microdomains have been linked to the cortical actin network [4], [5], [16], it is not surprising that a number of actin-associated proteins have been implicated in RSV assembly. Previous studies that have sought to elucidate the role of the cytoskeleton in the RSV life cycle have yielded ambiguous results, suggesting the role of cytoskeleton is complex. Gross disruption of the cytoskeleton using depolymerizing agents or inhibitors of polymerization affects viral replication [6]–[8], [17]. Both actin and profilin, an actin modulatory protein, act as transcription enhancing factors during RSV replication [7], [18], independent of actin polymerization [7], [19]. RSV infection activates RhoA, a kinase involved in actin cytoskeletal rearrangement [9], [20], and inhibition of RhoA results in a reduced number of blunted filaments and a shift to more spherical particle morphology [6], [21]. Finally, β-actin and a number of actin-related proteins have been found in the same sucrose gradient-purified fractions as RSV particles [10], [22].

We previously showed that the cytoplasmic tail of F (FCT), and specifically a phenylalanine residue three residues from the C terminus of FCT, is critical for viral assembly into filaments. The mechanism by which the FCT coordinates viral assembly and budding is not clear. In the current study, we first sought to examine whether the FCT facilitated viral assembly by interacting with host factors. We also sought to determine the mechanism by which disruption of ARE function affects viral assembly, specifically asking if this disruption robbed the cell surface of critical cytoskeletal elements needed for virion assembly or budding. Finally, we asked whether disruptions in actin or tubulin cytoskeletal dynamics affect viral assembly into filaments. The results suggest that although some cytoskeletal elements are peripherally associated with the assembly mechanism, RSV assembly into filaments using a mechanism that is independent of the host cytoskeleton and most actin-associated proteins. The data suggest that viral proteins are the principal mechanical factors driving the formation of the structures of viral particles at the cell surface. This finding is remarkable considering the length and structural complexity of the viral filaments.

## Results

### Filamin A (FLNA) Localizes to Viral Filaments

**Figure 1:**
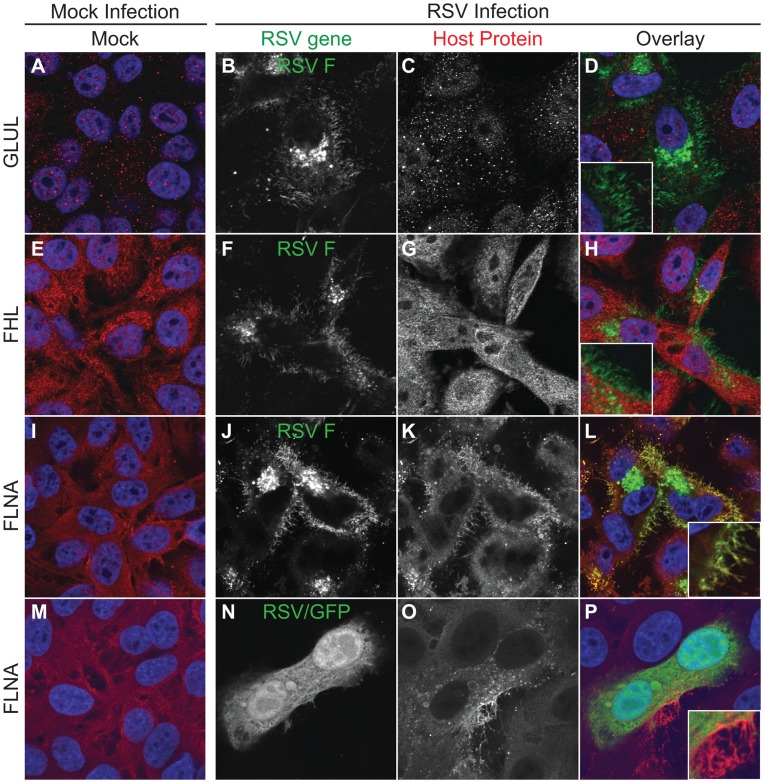
Cellular localization of candidate proteins from an FCT Y2H screen. HEp-2 cells were inoculated with RSV strain A2 at than MOI = 1.0 and incubated for 24 hours. RSV F and the indicated cellular proteins were detected by indirect immunofluorescence. Column 1 (panels A, E, I, and M) shows mock-infected cells, and columns 2–4 show RSV infected cells. Column 2 (panels B, F, J, and N) shows the indicated RSV protein; column 3 (panels C, G, K, and O) shows the indicated cellular proteins; and column 4 (panels D, H, L, and P) shows the overlay with the RSV protein in green and the cellular protein in red.

Since we had previously shown that the FCT is essential for viral assembly, we sought to determine if host factors play a role in viral filamentous assembly though interactions with the FCT. We performed a yeast two-hybrid screen using a custom human lung tissue cDNA library and probed with a peptide corresponding to the *wt* FCT as bait. The results from this screen are summarized in Table S1. We then sought to screen candidate FCT-interacting proteins by microscopy to determine if any localized to viral structures in infected cells. HEp-2 cell culture monolayers were either mock-infected or infected with *wt* RSV and incubated for 24 hours in complete growth medium. Cells then were fixed and immunostained for RSV F to mark viral filaments and the indicated host protein. Figure 1 shows the localization of these proteins in mock- or RSV-infected cells. Several did not localize to filaments. Glutamate-ammonia ligase (GLUL) was seen throughout the cell in punctate granules, but did not localize to viral filaments in RSV-infected cells (Fig. 1A–D). Four and a half LIM domains 2 (FHL2) protein exhibited a diffuse pattern throughout the cytoplasm, but it also failed to co-localize with RSV F in viral filaments (Fig. 1E–H). In contrast, although filamin A also localized throughout the cytoplasm, it specifically co-localized with RSV F in viral filaments (Fig. 1I–L).

To confirm this co-localization, HEp-2 cell culture monolayers were mock-infected or infected with *wt* RSV that also encodes a GFP protein (RSV/GFP) in order to mark infected cells (Fig. 1M–P). Compared to neighboring uninfected cells, filamin A in infected cells appeared to localize brightly to virus-induced structures at the cell surface that were kinked and clustered in a manner consistent with viral filaments. GFP, which typically diffuses throughout the cytoplasm when expressed in cells, was excluded from these structures, indicating that protein sorting into viral filaments is specific and cytoplasmic diffusion alone is not sufficient for filamin A incorporation into viral assembly structures.

### Actin-related Proteins are Mostly Excluded from Viral Filaments

Filamin A is an actin crosslinking protein that often anchors membrane glycoproteins to the actin cytoskeleton. Since we found filamin A localized to viral filaments, we reasoned that other actin-associated proteins in the apical domain of the cell might localize to viral filaments. Previously, we had found that disruption of the function of the apical recycling endosome (ARE) reduced RSV assembly and budding, but did not interrupt trafficking of viral proteins to the cell surface [3], [8]. Those findings may have suggested that inhibition of ARE function robbed the cell surface of host proteins required for assembly and budding. Therefore, we sought to define what host proteins were retained in the ARE when critical ARE motor or related proteins were inhibited. We developed a fine-resolution isolation procedure combining gradient isolation and fluorescence activated vesicle sorting (FAVS) for isolating constituents of the ARE, summarized in Figure S1. We expressed an EGFP-chimera of a dominant-negative inhibitor of myosin-Vb (EGFP-myosin Vb tail), and then physically sorted the resulting myosin-Vb tail inhibited recycling endosome (designated MIRE) vesicles. Gradient fractions from iodixanol gradients to identify MIRE vesicles were selected (fractions 6 through 10, indicated by red box in Figure S2), and then fraction contents were sorted physically using a sorting cytometer (Figure S3). We then performed tandem mass spectrometry on those MIRE vesicles to determine the major proteins retained in the ARE during inhibition of myosin Vb function and thus during inhibition of RSV assembly and budding. These proteomic studies revealed that the MIRE compartment contained peptides consistent with the presence of a number of host proteins (Figure S4, which shows MIRE proteins definitively identified in at least two of five independent experiments). The findings included a number of important actin-related proteins, such as ezrin, moesin, annexin A2, ezrin-radixin–moesin–binding phosphoprotein 50 (EBP50), α-actinin 1 and α-actinin 4. We therefore tested whether any of the actin-associated proteins localized to viral filaments in infected cells.

**Figure 2:**
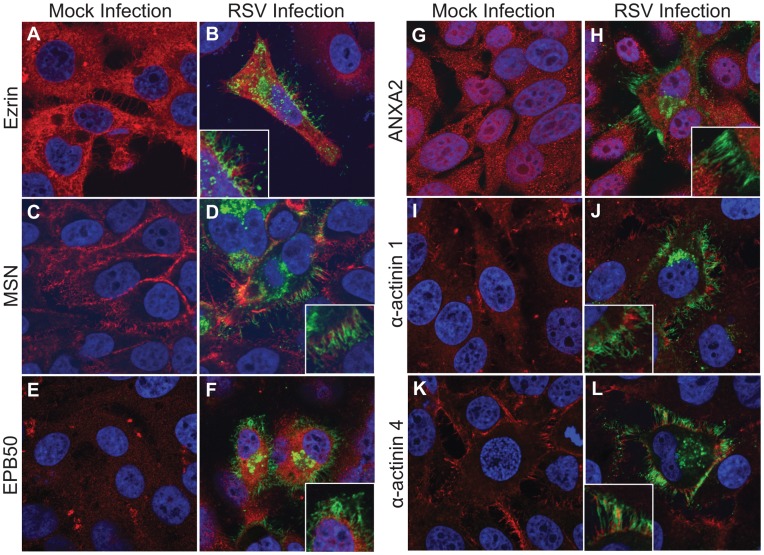
Cellular localization of cytoskeletal proteins found in MIRE vesicles. HEp-2 cells were inoculated with RSV stain A2 at an MOI = 1.0 and incubated for 24 hours. RSV F and the indicated cellular proteins were detected by indirect immunofluorescence. Columns 1 and 3 (panels A, C, E, G, I, and K) show mock-infected cells, while columns 2 and 4 (panels B, D, F, H, J, and L) show RSV infected cells. RSV F is shown in green and the indicated cellular protein is shown in red.

HEp-2 cell culture monolayers were either mock-infected or infected with *wt* RSV and incubated for 24 hours in complete growth medium. Figure 2 shows the localization of these proteins in mock- or RSV-infected cells. Ezrin, a member of the ezrin, radixin and moesin (ERM) family of proteins, localized diffusely through the cytoplasm and to membrane protrusions at the cell surface (Fig. 2A). These protrusions, however, were distinct from viral filaments that form at the cell surface in RSV-infected cells (Fig. 2B). Moesin, another member of the ERM family, localized largely underneath the plasma membrane and also to membrane protrusions (Fig. 2C). Like ezrin, moesin did not co-localize with RSV F in viral filaments, but remained in distinct host structures (Fig. 2D). EBP50, a scaffold protein that connects membrane proteins to ERM proteins, localized diffusely throughout the cytoplasm (Fig. 2E), but was excluded from viral filaments (Fig. 2F). Annexin A2 is another protein that has been reported to link membrane glycoproteins to the actin cytoskeleton. Although this protein localized diffusely throughout the cell (Fig. 2G), it did not localize to viral filaments (Fig. 2H). Finally, we examined two α-actinin proteins that are members of the large and diverse spectrin family of proteins. The α-actinin 1 and 4 proteins, which are expressed in non-muscle cells, are actin-binding proteins that are involved in actin crosslinking and also may mediate attachment of the plasma membrane to the actin cytoskeleton, much like filamin A. While α-actinin 1 was excluded from viral filaments completely (Fig. 2I–J), α-actinin 4 showed some minor co-localization with viral filaments, with variation from cell to cell (Fig. 2K–L). These data suggest that most actin-associated proteins are excluded from viral assembly structures, again providing evidence for specific sorting of proteins into viral filaments and exclusion of host proteins including cytoskeletal elements.

#### Knockdown of filamin A gene expression does not affect viral replication

**Figure 3:**
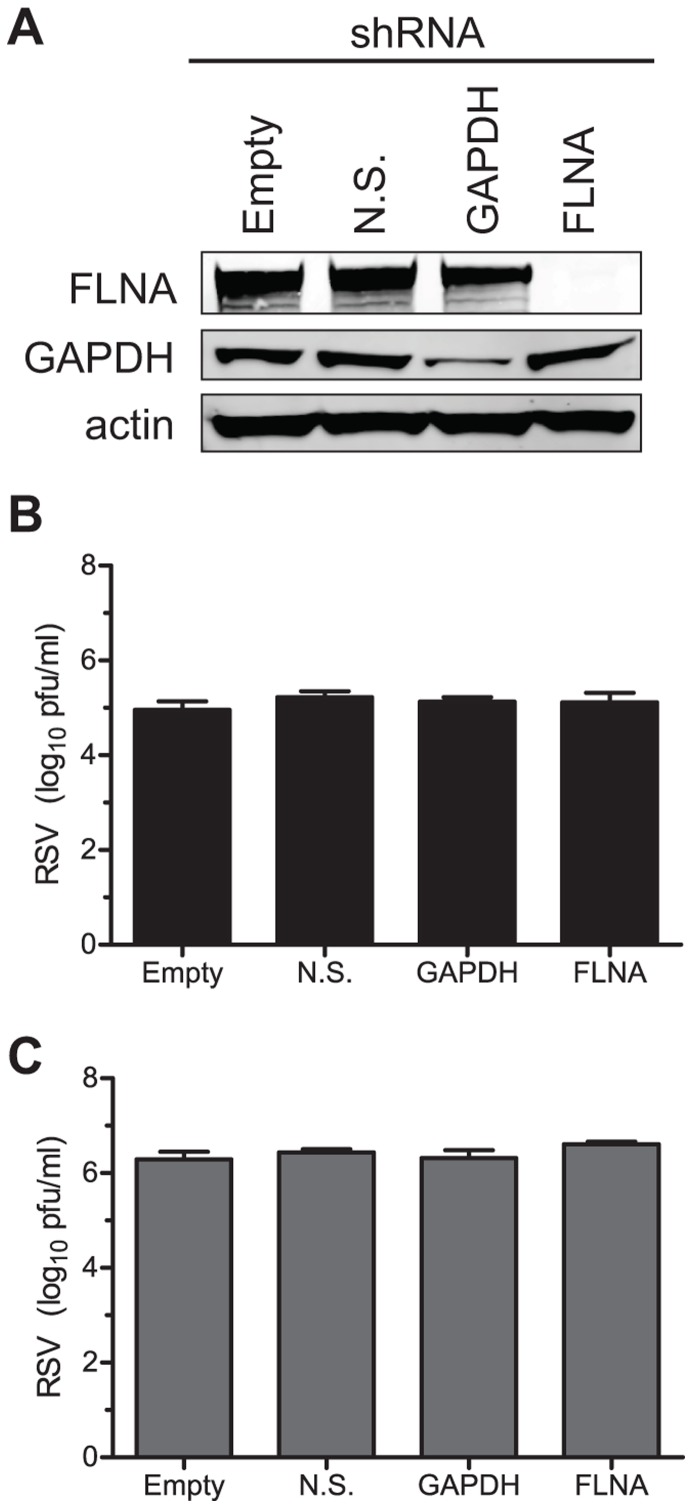
Viral titers in FLNA knockdown cells. HEp-2 cells were transduced with a lentivirus encoding an shRNA directed against the indicated protein. After selection, cell lysates were harvest and immunoblotted for the presence of filamin A, GAPDH, or actin (A). The same cells then were infected with RSV stain A2 at an MOI = 0.05 for 72 hours. Both cell-associated (B) and supernatant virus (C) yields were quantified by a plaque assay.

Since filamin A exhibited the greatest degree of localization with viral filaments of any cytoskeleton related proteins examined, we studied its role in assembly and budding further. We inhibited expression of filamin A to determine if reduction of expression inhibited the efficiency of viral replication in infected cells. To reduce filamin A gene expression, we generated lentiviruses that express an shRNA hairpin directed against filamin A. HEp-2 cell culture monolayers then were transduced with the filamin A shRNA lentivirus (FLNA), or with control lentiviruses encoding no shRNA (empty), an shRNA that is non-silencing (N.S.), or an shRNA directed against the housekeeping gene GAPDH. Cells were selected for shRNA expression using puromycin, and the resulting puromycin-resistant cell line was screened for protein knockdown and viral replication. Figure 3A shows that transduction of cells with the filamin A shRNA lentivirus caused a >99% knockdown of expression of the protein. As a parallel control, GAPDH expression was inhibited in cells transduced with GAPDH shRNA. Transduction with empty vector or non-silencing lentiviruses did not affect the expression of either GAPDH or filamin A. Since the knockdown of filamin A was efficient, we next infected these lentivirus-transduced cells with *wt* RSV strain A2 at an MOI = 0.05 to determine the effect of filamin A knockdown on RSV replication at 72 hours post-infection (h.p.i). Figures 3B and 3C show that neither supernatant nor cell-associated viral titer, respectively, was affected by the knockdown of filamin A. In addition to filamin A, we also tested whether other proteins from the yeast two hybrid FCT interaction screen were required for efficient viral replication by expressing shRNA against each protein delivered by lentiviruses, as described for filamin A. Expression of shRNA was confirmed by GFP expression from a second reading frame, but total viral yield at 72 h.p.i. was not affected (Table S2). Collectively, these data suggest that although filamin A protein localizes to viral filaments, it is not required for viral replication.

### Cytoskeletal Rearrangement is not Necessary for Viral Assembly into Filaments

**Figure 4:**
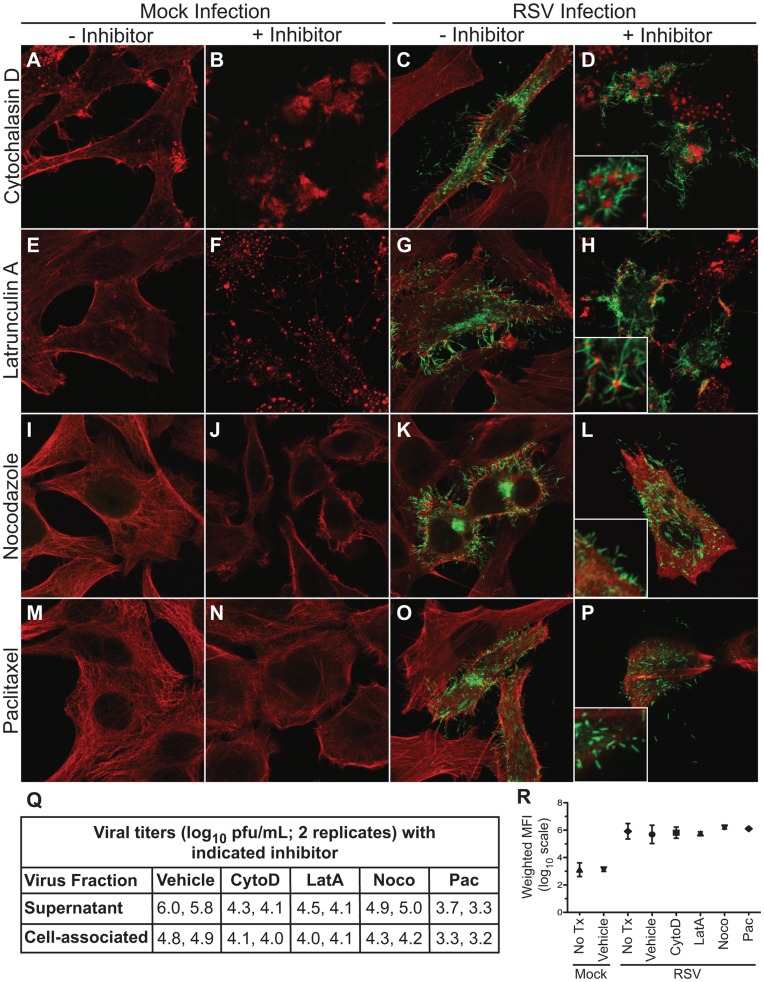
Effect of cytoskeletal inhibitors on viral assembly. HEp-2 cells were inoculated with RSV *wt* strain A2 at an MOI = 1.0 and incubated for 24 hours with medium containing either vehicle of the indicated inhibitor. At 24 hours cells were fixed, and RSV F, actin, and tubulin were detected by indirect immunofluorescence. Column 1 (panels A, E, I, and M) shows mock-infected cells in the presence of vehicle. Column 2 (panels B, F, J, and N) shows mock-infected cells in the presence of the indicated inhibitor. Column 3 (panels C, G, K, and O) shows RSV infected cells in the presence of vehicle. Column 4 (panels D, H, L, and P) shows RSV infected cells in the presence of the indicated inhibitor. Panel Q: HEp-2 cells were infected with RSV at an MOI = 0.1 for 72 hours. Supernatant and cell-associated fractions were collected in duplicate and quantified using a viral plaque assay. Viral yields are presented in log_10_ scale. Panel R: total surface expression of RSV F was determined by flow cytometric analysis in mock-infected or RSV-infected cells (MOI = 3.0) treated with vehicle or the indicated inhibitor for 24 hours. Data are plotted as mean, and error bars represent standard deviation. Weighted mean fluorescence intensity (MFI) is MFI multiplied by the frequency of positive cells.

Since actin-associated proteins were mostly excluded from viral filaments and may not affect viral replication, we next sought to determine whether cytoskeletal polymerization is necessary for viral assembly into filaments. We had determined previously the inhibitor concentrations that resulted in maximum effect on the cytoskeleton with the least cellular toxicity [11], [17]. The effect of inhibitor concentrations on virus replication was determined to be similar to the previously reported results, showing 0.95 to 2.4 log_10_ pfu/mL reduction in supernatant associated virus and 0.6 to 1.6 log_10_ pfu/mL reduction in cell-associated virus (Figure 4Q).

Using the same conditions, we asked whether or not gross cytoskeletal disruptions affected viral assembly into filaments. HEp-2 cell culture monolayers were inoculated with *wt* RSV strain A2, and then grown in growth medium containing the indicated inhibitor or vehicle for 24 hours. Cells then were immunostained for RSV F protein to mark viral filaments and for actin or tubulin, depending on which cytoskeletal function was disrupted. Remarkably, viral assembly into filaments at the cell surface was not inhibited when actin or tubulin polymerization was disrupted (Fig. 4A–P). Cells treated with cytochalasin D (Fig. 4B and 4D) or latrunculin A (Fig. 4F and 4H) showed a lack of cortical actin structure underneath the plasma membrane. However, this disruption did not affect the formation of viral filaments (Fig. 4D and H). Viral filaments, however, often appeared to originate from depolymerized actin aggregates (highlighted by the insets in Fig. 4D and 4H). These images suggested that although actin polymerization is not necessary for viral filament formation, actin or actin related proteins in these aggregates may play some peripheral role in filament initiation or anchoring. Inhibition of tubulin polymerization using nocodazole (Fig. 4I-L) or depolymerization of tubulin using paclitaxel (Fig. 4M-P) also did not affect viral assembly into filaments. Finally, we determined if F protein surface expression was altered by disruption of the cytoskeleton, using flow cytometric analysis (Fig. 4R). F protein surface expression was not altered in the presence of inhibitors, suggesting that cytoskeletal rearrangements are not necessary for F protein expression or trafficking to the cell surface. These data collectively indicate that viral filaments are able to initiate membrane deformation and to elongate by a virus-mediated mechanism that is distinct from that used by actin- and tubulin-based cellular protrusions.

## Discussion

The mechanism of RSV assembly and budding is of interest since it appears to use an unconventional pathway that is independent of host proteins of the multi-vesicular body [7], [12]. A number of viruses have been shown to coopt the use of host cytoskeleton and related proteins to accomplish viral assembly and budding [3], [23]. We sought to determine if RSV achieves its unique program using apical cytoskeleton or related proteins for assembly and budding. We had determined previously that the RSV FCT coordinates filament formation, and here we show data suggesting that the FCT interacts with the host protein filamin A. We found this interaction interesting because it seemed attractive to think that anchoring emerging viral filaments to the cytoskeleton might allow some mechanical advantage needed to deform host cell membrane and elongate the viral filaments. Indeed, some of the images in the setting of depolymerized actin suggested that the base of filaments might be transiently associated with actin. Comprehensive yeast two-hybrid and proteomic studies revealed that cytoskeletal elements are abundant in the apical region of polarized cells, but we found that the dominant apical cytoskeleton associated proteins did not localize to filaments. Even filamin A, the sole host protein that we confirmed to be located reliably in filaments, was not required for viral replication, suggesting that an FCT-filamin A interaction is not necessary for viral assembly into filaments. Finally, we showed that viral filamentous assembly did not depend on host cell ability to rearrange the actin or tubulin cytoskeleton, indicating that the mechanisms by which RSV assembles into viral filaments is independent of host cytoskeletal rearrangement.

Although we and others have reported that actin associated proteins localize to viral assembly structures [7], [22], it is remarkable that viral assembly into long, complex filamentous structures that require the manipulation of the plasma membrane can occur without the need for actin polymerization. The data suggest that viral constituents achieve complex tasks at the membrane, including specific sorting of viral proteins into the filaments while excluding most host proteins, extrusion of long membranous structures from the cell without a cytoskeletal infrastructure, incorporation of nucleocapsids, and scission of the membrane to release infectious particles. It seems clear that viral elements provide the dynamic mechanical force to initiate and extend viral filaments, not polymerization of the cytoskeleton. However, the findings that inhibition of actin polymerization is not required for assembly and budding does not completely exclude the possibility that the cortical actin network or associated proteins still function in an accessory structural role for viral replication. We and others have shown previously that actin polymerization is necessary for optimal viral replication [17], [24], [25], and our observation of multiple viral filaments radiating from actin aggregates under depolymerizing conditions also may support the hypothesis that actin plays an accessory structural role in replication. In fact, other groups have described actin at the base of viral filaments [25], and β-actin has been described to interact with RSV N [26]. Some investigators have reported β-actin in viral particles [22], [26]. Furthermore, actin associated pathways are important for viral filamentous assembly [21], [27]. Finally, actin enhances viral transcription, although its role as a transcription factor does not rely on the ability to polymerize [18].

Since actin does not provide the physical infrastucture for a viral filament, the structure for a complex, kinked viral filament may be determined by features of viral proteins themselves. We have shown previously that the four major structural proteins are required for filament formation independent of viral infection, and viral filaments are distinct from actin-based cellular structures [8]. Similarly, a recent study looking at viral filaments by scanning electron microscopy showed that in the absence of the M protein, only short filament-like structures with F and G were observed on the cell surface [12]. These data may suggest that while the viral glycoproteins are sufficient for the initial outward bud formation, the structure of the filament is driven by the M protein. The matrix proteins of other viruses have been shown to oligomerize into higher order structures [28] and it may be that oligomerization of the RSV M protein is responsible for elongation of the structure of viral filaments.

The remarkable finding of this study is that complex, structured RSV filaments form apparently normally even when actin or microtubule function polymerization is completely inhibited. There is some precedence for a dominant role of viral proteins in controlling the complex formation of filamentous virion particles, especially in studies of influenza virus. An interesting feature of influenza A virus budding is that most strains of the virus form two distinct types of virions: spherical particles approximately 100 nm in diameter and much longer filamentous particles. The influenza M1 protein appears to be the principal determinant of particle shape [29], [30]. Influenza differs from RSV, however, in that budding of spherical influenza particles occurs in the absence of an intact actin cytoskeleton but influenza filament formation does not [31], [32]. Further studies of the host and viral molecular determinants of filament formation for these viruses are warranted.

## Materials and Methods

### Cell Culture and *wt* RSV Virus Preparations

HEp-2 cells (ATCC CCL-23) were maintained in OPTI-MEM I medium (Invitrogen) containing 2% (v/v) fetal bovine serum (FBS), 1% (v/v) L-glutamine, 2.5 µg/mL amphotericin B, and 1% (v/v) penicillin-streptomycin. HEK293T cells (ATCC CRL-11268) were maintained in DMEM/F12 medium containing 10% FBS, 1% (v/v) L-glutamine, 2.5 µg/mL amphotericin B, and 1% (v/v) penicillin-streptomycin. Vero cells were maintained in EMEM 10% FBS and 1% (v/v) penicillin-streptomycin. The Madin-Darby canine kidney (MDCK) T23 cell line (Clontech) was co-transfected with cDNA for myosin Vb-tail cloned into the pTRE2 expression vector and pCB7. Transfected cell clones were selected by growth in the presence of hygromycin (150 µM). Induction and suppression of GFP fusion protein expression was tightly regulated by the absence or presence of doxycycline. These cell lines were maintained in DMEM and Ham’s F-12 medium (Invitrogen) supplemented with 10% FBS, 320 µg/mL L-glutamine, 1% (vol/vol) nonessential amino acids, 2.7 µg/mL amphotericin B, and 45 µg/mL gentamicin. Additionally, media used for the myosin Vb-tail cell lines were supplemented with 20 ng/mL of doxycycline as needed, to repress plasmid-based protein expression. The RSV *wt* strain A2 was expanded in HEp-2 cells, and RSV/GFP (kindly provided by MedImmune) was expanded in Vero cells.

### Cytoskeletal Inhibitors

HEp-2 cells were treated with actin or tubulin inhibitors, as previously described [17]. Briefly, cytochalasin D (Sigma) was prepared at 2 mg/ml in ethanol; latrunculin A (Enzo Life Sciences) at 1 mg/ml in ethanol; nocodazole (Sigma) at 10 mg/ml in DMSO; and paclitaxel (Sigma) at 25 mg/ml in ethanol. Inhibitor concentrations were previously optimized for maximum effect by cell morphology with the least amount of toxicity. For immunostaining, HEp-2 cell monolayers on 12 mm micro cover glasses (VWR, No. 2) were inoculated at an MOI = 1.0 and incubated with medium containing either vehicle or the indicated inhibitor for 24 hr.

### RSV Infections and Viral Titration

For quantification of viral yield in the presence of inhibitors and in knockdown cell lines, HEp-2 cell monolayers were inoculated at an MOI = 0.05. Cells were then washed and medium was added containing either vehicle or indicated inhibitor for the cytoskeletal inhibitor assays or normal media for knockdown cell lines. At 3 days post infection, cell supernatant was harvested and normalized for volume. The samples were then clarified of cellular debris by centrifugation in a microcentrifuge at 13,000 rpm for 10 min. The resulting supernatant was designated as supernatant virus. The cell monolayer was resuspended in an equal volume of medium, scraped, and freeze/thawed 3× using a dry ice/ethanol bath and a 37°C water bath. The samples then were clarified of cellular debris by centrifugation in a microcentrifuge at 13,000 rpm for 10 min. The resulting solution was designated cell-associated virus.

### Fixation and Immunostaining

HEp-2 cells were mock infected or infected with RSV A2 *wt* strain or RSV/GFP at an MOI = 1.0 for 24 hours. Cells then were fixed with 3.7% (w/v) paraformaldehyde in phosphate buffered saline (PBS) for 10 min. Cells were permeabilized with 0.3% (w/v) Triton X-100 and 3.7% paraformaldehyde in PBS for 10 min at RT. After fixation, cells were blocked in 3% (w/v) BSA in PBS for 60 min followed by addition of primary antibodies in the blocking solution for 60 min. Cells then were washed three times in PBS, and species-specific IgG Alexa Fluor (Invitrogen) was added at a dilution of 1∶1,000 in block solution for 60 min to detect primary antibodies. Cells were washed three times in PBS and fixed on glass slides using Prolong Antifade kit (Invitrogen). All steps were performed at room temperature (RT). Images were obtained on a Zeiss inverted LSM510 confocal microscope using a 63×/1.40 Plan-Apochromat oil lens. An anti-RSV F protein humanized mouse monoclonal antibody (palivizumab; MedImmune) was obtained from the Vanderbilt Pharmacy and used to visualize RSV F along with a goat anti-human Alexa Fluor secondary antibody (Invitrogen). F-actin was visualized using rhodamine phalloidin (Invitrogen), and TO-PRO-3 iodide was used to visualize the nucleus (Invitrogen). Tubulin, GLUL, FHL, FLNA, ezrin, MSN, ANXA2, EBP50, α-actinin 1, and α-actinin 4 were visualized using primary antibodies obtained from Abcam and species-specific Alexa Fluor secondary antibodies (Invitrogen).

### Flow Cytometric Assay for Quantitative Surface Expression of RSV F

HEp-2 cells were inoculated with RSV for 1 hour at 37°C at an MOI = 3.0. The inoculum then was removed and each inhibitor was added, as described above. After 24 hours, cells were treated with 20 mM EDTA in PBS to form a single cell suspension. Cells were washed two times in wash buffer (2% FBS in PBS) and then incubated with palivizumab at 1 µg/mL for 30 min at RT. An Alexa Fluor goat anti-mouse 488 secondary antibody suspension was used at a final concentration of 2 µg/mL. Cells were analyzed on a five-laser custom LSR II flow cytometer (Becton Dickinson) in the Vanderbilt Medical Center Flow Cytometry Shared Resource. Data analysis was performed using FlowJo (version 7.6.1). Weighted mean fluorescent intensity (MFI) was calculated by multiplying the raw mean fluorescent intensity by the frequency of positive cells. Statistical analysis was performed using a Student’s *t-test* of three independent experiments. P values less than 0.05 were considered significant.

### Identification of Proteins that Interact with RSV F Protein Cytoplasmic Tail by Yeast Two-hybrid (Y2H) Screening

Bait cloning and Y2H screening were performed by Hybrigenics, S.A., Paris, France. A cDNA encoding the RSV strain A2 F cytoplasmic tail was synthesized and then PCR-amplified and cloned in a Y2H vector optimized by Hybrigenics. The bait construct was checked by sequencing the entire insert, and was subsequently transformed in the L40Δ GAL4 yeast strain [33]. Fresh human lung tissue was obtained from the healthy margins of a lung resection, and then disrupted and total RNA extracted. A human normal lung random-primed cDNA library containing ten million independent fragments was generated then transformed into the Y187 yeast strain and used for mating. High mating efficiency was obtained by using a specific mating method [34]–[36]. The screen was first performed on a small scale to adapt the selective pressure to the intrinsic property of the bait. Neither toxicity nor auto-activation of the bait was observed. Then, the full-scale screen was performed in conditions ensuring a minimum of 50 million interactions tested, in order to cover five times the primary complexity of the yeast-transformed cDNA library [37]. 270 million interactions were tested for interaction with RSV F cytoplasmic tail. After selection on medium lacking leucine, tryptophan, and histidine, 45 positive clones were picked, and the corresponding prey fragments were amplified by PCR and sequenced at their 5′ and 3′ junctions. Sequences then were filtered and constructed as contigs, as described previously [38], and compared to the latest release of the GenBank database using BLASTN [39]. A Predicted Biological Score (PBS) was attributed to assess the reliability of each interaction, as described previously [38]. Briefly, the PBS relies on two different levels of analysis. First, the local score took into account the redundancy and independency of prey fragments, as well as the distributions of reading frames and stop codons in overlapping fragments. Second, the global score took into account the interactions found in all the screens performed at Hybrigenics using the same library. In addition, potential false-positives were flagged by a specific “E” PBS score. This was done by discriminating prey proteins containing “highly connected” domains, previously found several times in screens performed on libraries derived from the same organism. The PBS scores have been shown to correlate positively with the biological significance of interactions [37], [40].

### Endosome Isolation

MDCK cells expressing EGFP-myosin Vb Tail were grown on 44 cm^2^ Transwell dishes with 0.4 µm pore (Costar). Cells were grown at confluence for five days, and then were scraped and collected on ice then pelleted and resuspended in homogenization buffer, 0.32 M sucrose, 1 mM EDTA, 10 mM Tris-HCl, pH 7.4. Cells were sheared by passing them through a sequential series of 18 to 23 gauge needles. The cell lysate then was centrifuged differentially at 1,000, 5,000, 15,000, and 100,000×*g*. The 100,000×*g* pellet was stained with the membrane dye DiD (Invitrogen) for 20 minutes at 37°C. This preparation then was centrifuged on a 5–40% step gradient of iodixanol (Sigma) for 18 hours at 90,000×*g*. Fractions were collected and were physically sorted on a flow cytometer.

### Fluorescence-activated Vesicle Sorting (FAVS)

Sorting was performed on a custom FACSAria sorting cytometer (Becton Dickinson) modified with a forward scatter PMT, and standardized for linearity and sensitivity using 8 peak beads (Spherotech, Lake Forest, IL), as previously described [41]. Briefly, resolution of particle size was refined using green fluorescent beads ranging in size from 40 nm–700 nm (Duke Scientific, Fremont, CA). A high salt sheath, 78 mM KCl, 4 mM MgCl, 8 mM CaCl, 10 mM EGTA and 50 mM HEPES, pH 7.0, was filtered through a 100 nm filter prior to installation into the Aria sheath reservoir. Two additional inline filters (200 nm) were used to assure the sheath had the lowest background possible. Unstained and single-stained (DiD or GFP only) vesicles were used to compensate for spectral overlap. Double positive (DiD and EGFP-myosin Vb) vesicles were selected by gating and subjected to pulse processing analysis for doublet discrimination. Extensive preliminary studies were performed to determine and validate the sorting gates.

### LC-MS-MS Analysis and Protein Identification

Double-positive sorted endosomes then were pelleted at 120,000×*g*. This pellet was resuspended in 2X NuPAGE LDS sample buffer with reducing agent (Invitrogen), and samples were heated for 15 min at 85°C, and then applied to a 10% NuPAGE Bis-Tris gel (Invitrogen). Electrophoresis continued until the dye front had migrated into the gel ∼1–2 cm. The gel then was stained with Bio-Safe Coomassie (Bio-Rad), and the protein band was excised from the gel, minced, and washed. Proteins were subjected to in-gel trypsin digestion [42], and the resulting peptides were eluted from the gel and submitted for LC-MS-MS analysis. LC-MS-MS analysis of the resulting peptides was performed using a Thermo Finnigan LTQ ion trap mass spectrometer equipped with a Thermo MicroAS autosampler and Thermo Surveyor HPLC pump, Nanospray source, and Xcalibur 1.4 instrument control. Peptides were separated on a packed capillary tip (100 µm x 11 cm) with C18 resin (Monitor C18, 5 µm, 100 Å, Column Engineering, ON, Canada) using an in-line solid-phase extraction column that was 100 µm x 6 cm packed with the same C18 resin [using a frit generated with liquid silicate Kasil 1 [43] similar to that previously described [44] except the flow from the HPLC pump was split prior to the injection valve. The flow rate during the solid phase extraction phase of the gradient was 1 ml/min and during the separation phase was 700 nL/min. Mobile phase A was 0.1% formic acid, and mobile phase B was acetronitrile with 0.1% formic acid. A 95-min gradient was performed with a 15-min washing period (100% A for the first 10 min followed by a gradient to 98% A at 15 min) to allow for solid phase extraction and removal of any residual salts. After the initial washing period had passed, a 60-min gradient was performed where the first 35 min was a slow, linear gradient from 98% to 75% A, followed by a faster gradient to 10% A at 65 min and an isocratic phase at 10% A to 75 min. MS-MS spectra of the peptides were performed using data-dependent scanning in which one full MS spectrum, using a full mass range of 400–200 atomic mass units, was followed by 3 MS-MS spectra. In addition to this one-dimensional LC-MS analysis, peptides were also subjected to two-dimensional LC-LC-MS analysis in which the peptides were first fractionated using strong cation exchange chromatography (100 µm×7 cm Luna SCX column, Phenomonex, Torrance, CA) using a 0–500 mM (pH 3.0–8.0) ammonium formate gradient in 25% acetonitrile as described previously [45] except a final salt bump of 0.5 M ammonium formate during the last 10 min of the 65 min was substituted for the KCl bump. After collection of the flowthrough fraction, each of the nine separate fractions was collected, the last three fractions were combined, and each fraction (including the flow through fraction) was subjected to a reverse-phase separation directly inline with the LTQ as described above. Proteins were identified using the cluster version of the SEQUEST algorithm [46] using the human subset of the Uniref 100 database (www.uniprot.org). The database was concatenated with the reverse sequences of all the proteins in the database to allow for the determination of false positive rates. Protein matches were preliminarily filtered using the following criteria: if the charge state of the peptide is 1, the cross-correlation score (xcorr) is ≥1, the ranking based on preliminary score (RSp) is ≤5, and the preliminary score (Sp) is ≥350. If the charge state is 2, the xcorr is ≥1.8, the RSp is ≤5, and the Sp is ≥350. If the charge state is 3, the xcorr is ≥2.5, the RSp is ≤5, and the Sp is ≥350. Once filtered based on these scores, all protein matches that had less than two peptide matches were eliminated. These filtering criteria achieved a false positive rate of <1% in all datasets. Several preliminary analyses were performed as a matter of methods development, then five completely independent large-scale vesicle isolation and proteomic analysis experiments were performed to acquire the final proteomic data.

### Generation of shRNA Lentiviruses and Cell Populations with Knockdown of Gene Expression

Human GIPZ lentiviral shRNAmir constructs were obtained from the Vanderbilt Genomic Sciences Resource Core (filamin A (FLNA), alpha clone V2LHS_131780, positive GAPDH control, and negative non-silencing and empty vector controls). Lentiviruses were produced using the Open Biosystems Trans-Lentiviral GIPZ Packaging system (Thermo Scientific) in HEK293T cells according to kit instructions. Briefly, cells were transfected with pGIPZ lentiviral vector and packaging mix. Supernatant was collected at 48 and n 72 hours post transfection, clarified at 3,000 rpm for 20 min at 4°C, and filtered using a 0.45 µm PES filter unit. Filtered supernatant was concentrated using ultracentrifugation at 23,000 rpm for 2 hours. Pellet was resuspended in serum-free DMEM. Transduction efficiency was determined using flow cytometry and GFP expression levels in HEp-2 cells in the presence of 10 µg/mL polybrene (Millipore). Transduced HEp-2 cells were selected for expression using 5 µg/mL puromycin. Expression levels and viral titers were determined from the resulting heterogeneous population.

### Quantitative Analysis of Total Cell Lysate in Knockdown Populations

HEp-2 cells transduced with indicated lentivirus were grown under puromycin selection. Cell lysates were harvested using a single detergent lysis buffer (50 mM Tris-HCl, 150mM NaCl, 1% Triton X-100, pH 8.0) containing 1∶200 dilution of mammalian protease inhibitor cocktail (Sigma). Lysates were separated on 4–12% NuPAGE Bis-Tris gels and transferred to nitrocellulose membranes (Invitrogen). Membranes were blocked for one hour using Odyssey blocking buffer (Li-Cor) diluted 1∶1 in PBS. Primary antibodies for β-actin (Abcam, 1∶5,000), FLNA (Abcam, 1∶500), or GAPDH (Millipore, 1∶500) were diluted in blocking buffer diluted 1∶1 with PBS+0.1% Tween-20. Secondary antibodies were diluted at 1∶5,000 (goat anti-mouse IRDye 800CW and donkey anti-goat IRDye700, Li-Cor) in blocking buffer. Bands were imaged using the Odyssey Infrared Imaging System.

## Supporting Information

Figure S1 **Strategy for isolation of myosin Vb tail inhibited recycling endosome (MIRE) vesicles.** After cells were polarized and expressing myosin Vb-tail, the cells were collected and sheared, followed by organelle differential fractionation by size. The 100 k×g pellet was collected and all membrane-bound structures were then labeled with the lipid dye DiD. These vesicles were then separated by density centrifugation. Relevant fractions were collected and pooled for further purification through fluorescence-activated sorting. The proteins in the resulting sorted vesicles were in-gel digested and applied to a LC MS/MS mass spectrometer to generate tandem mass spectra and subsequently a list of proteins.
(PDF)

Figure S2 **Analysis of cellular fractions from iodixanol gradients to identify MIRE vesicles.** When the density gradient was analyzed for the myosin Vb-tail endosomes, we observed three peaks of myosin Vb localization. The first fraction of myosin Vb vesicles floated to the top of the gradient between fractions 6–10. The two additional peaks were located at fractions 18 and 25. Yet the latter two peaks were overlapping with the ER and other endosome components. When the density fractions were analyzed for the ER marker calnexin, the ER was found to localize across nearly the entire gradient peaking at fraction 18. A marker for the transcytotic pathway, dIgA, also showed three peaks. All three peaks are similar in localization to that of myoVb-tail expression. The transcytotic pathway moves from the basolateral early endosome to the common endosome before arriving at the ARE. Because cells were loaded with dIgA at the basolateral membrane, it is thought that the three peaks on the western blots may correlate with these three organelles, and the peak at fraction 25 may be the basolateral endosome. We reasoned that the more contaminating cellular structures that can be removed, the more accurate the proteomic analysis would be. The total protein concentration and lipid marker DiD show that the least contamination occurred within the 6–10 fraction peak of myosin Vb-tail expressing cells. Because fractions 6–10 were the least contaminated, and contained both myosin Vb-tail and dIgA, we decided to pool these fractions for further purification through fluorescence-activated sorting.
(PDF)

Figure S3 **Flow cytometric sorting for final purification of MIRE vesicles.** Pooled gradient fractions were purified further through fluorescence-activated sorting. These pooled fractions were sorted through a series of gates based on the content of GFP, DiD and size to remove positive events that may be nonspecifically bound to another vesicle. The final sample contained 2% of the starting material, and showed greater than 90% specificity for GFP and DiD staining. When the pre- and post-sort material was analyzed by confocal microscopy, we observed little co-localization in the pre-sort material, whereas the post-sort material had high co-localization of both GFP and DiD.
(PDF)

Figure S4 **MIRE vesicle proteomics list.** The purified population of microsomes was reduced, denatured and subjected to SDS-PAGE. The sample was only electrophoresed about one cm into the gel, and Coomassie stained. This material that formed a smear then was excised, and proteins were in-gel digested and applied to a LC MS/MS mass spectrometer to generate tandem mass spectra and subsequently a list of proteins. High quality identification of proteins that occurred in two or more of five independent analyses (columns 1–5) are shown. Detection in the analysis is indicated by an X.
(PDF)

Table S1 **Summary of candidate proteins from FCT Y2H screen of a custom lung cDNA library.** A Y2H screen was performed by Hybrigenics (Paris, France) using the wt FCT peptide fused to LexA at the N-terminus as bait to screen a custom human lung cDNA library. The candidate proteins are listed by their gene name along with a description of the gene and associations. The cDNA fragment also was analyzed for being in frame (IF), unknown frame (??), and whether the interaction was with an antisense strand (antisense) or in the 3′ UTR (3′ UTR).
(PDF)

Table S2 **Summary of results available antibodies and shRNAs.** The available reagents for the FCT Y2H candidate genes are summarized. For genes with available antibodies, HEp-2 cells were inoculated with RSV wt strain A2 at an MOI = 1.0 for 24 hours. At 24 hours, cells were fixed, and RSV F and the indicated cellular protein were detected by indirect immunofluorescence. For genes with available shRNA lentivirus constructs, HEp-2 cells were transduced with each lentivirus construct. A heterogeneous population was selected using puromycin, and expression of the shRNA was confirmed by concomitant expression of GFP. Cells then were infected with RSV strain A2 at an MOI = 0.05 for 72 hours and both supernatant and cell associated virus yields were determined by plaque assay. The results for localization to viral filaments marked by RSV F and affect of shRNA expression on viral yields is summarized.
(PDF)
