# Supplementary material for: Respiratory Syncytial Virus Assembles into Structured Filamentous Virion Particles Independently of Host Cytoskeleton and Related Proteins
Source: PLoS One. 2012 Jul 13;7(7):e40826. doi: 10.1371/journal.pone.0040826 (PMC3396619; doi:10.1371/journal.pone.0040826)
Supplement: Figure S1 — Strategy for isolation of myosin Vb tail inhibited recycling endosome (MIRE) vesicles. After cells were polarized and expressing myosin Vb-tail, the cells were collected and sheared, followed by organelle differential fractionation by size. The 100 k×g pellet was collected and all membrane-bound structures were then labeled with the lipid dye DiD. These vesicles were then separated by density centrifugation. Relevant fractions were collected and pooled for further purification through fluorescence-activated sorting. The proteins in the resulting sorted vesicles were in-gel digested and applied to a LC MS/MS mass spectrometer to generate tandem mass spectra and subsequently a list of proteins. (PDF) [file pone.0040826.s001.pdf]

Figure S1. Strategy for isolation of myosin Vb tail inhibited recycling endosome (MIRE) vesicles

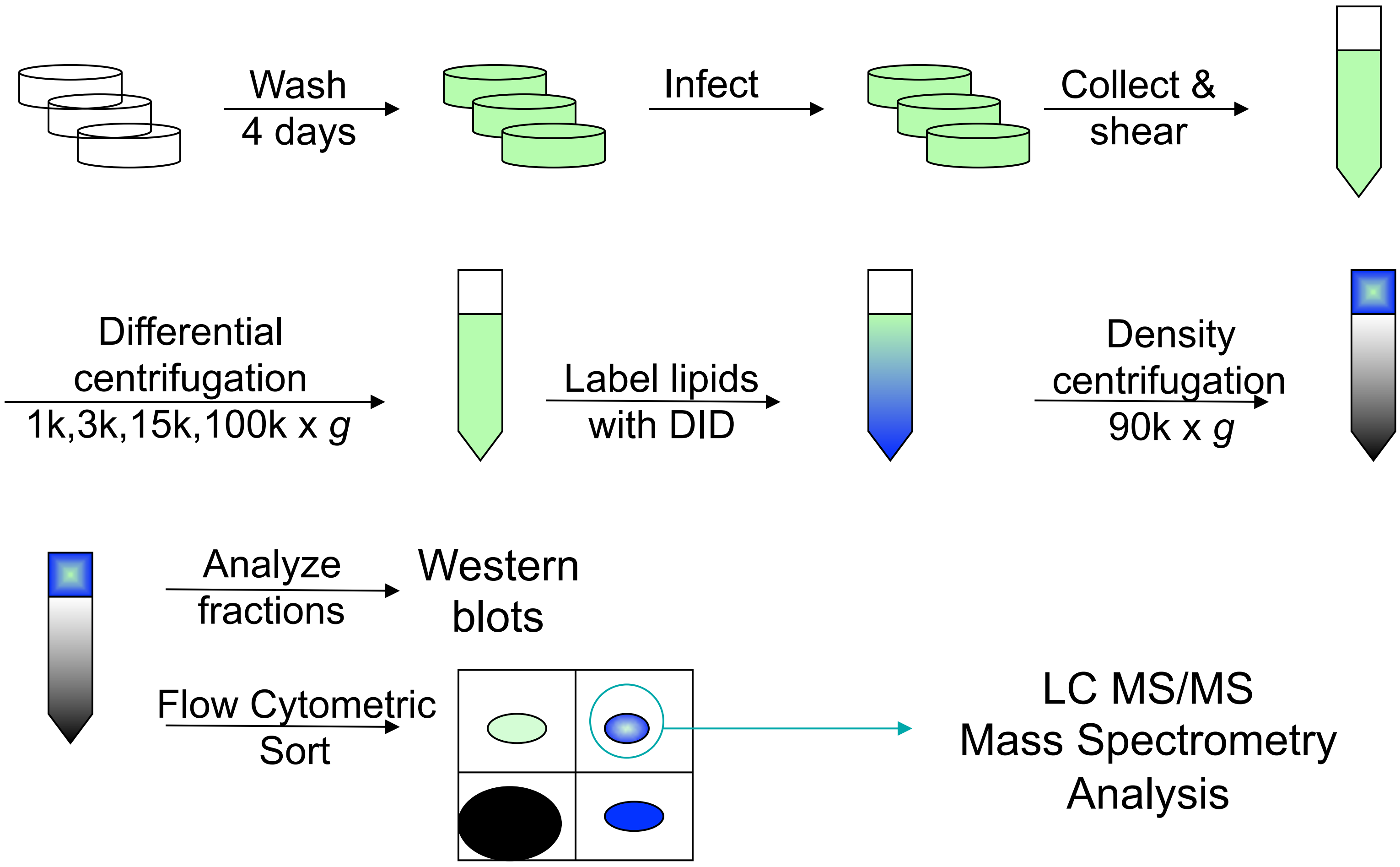

After cells were polarized and expressing myosin Vb-tail, the cells were collected and sheared, followed by organelle differential fractionation by size. The 100k x g pellet was collected and all membrane-bound structures were then labeled with the lipid dye DiD. These vesicles were then separated by density centrifugation. Relevant fractions were collected and pooled for further purification through fluorescence-activated sorting. The proteins in the resulting sorted vesicles were in-gel digested and applied to a LC MS/MS mass spectrometer to generate tandem mass spectra and subsequently a list of proteins.
