# Supplementary material for: Respiratory Syncytial Virus Assembles into Structured Filamentous Virion Particles Independently of Host Cytoskeleton and Related Proteins
Source: PLoS One. 2012 Jul 13;7(7):e40826. doi: 10.1371/journal.pone.0040826 (PMC3396619; doi:10.1371/journal.pone.0040826)
Supplement: Figure S2 — Analysis of cellular fractions from iodixanol gradients to identify MIRE vesicles. When the density gradient was analyzed for the myosin Vb-tail endosomes, we observed three peaks of myosin Vb localization. The first fraction of myosin Vb vesicles floated to the top of the gradient between fractions 6–10. The two additional peaks were located at fractions 18 and 25. Yet the latter two peaks were overlapping with the ER and other endosome components. When the density fractions were analyzed for the ER marker calnexin, the ER was found to localize across nearly the entire gradient peaking at fraction 18. A marker for the transcytotic pathway, dIgA, also showed three peaks. All three peaks are similar in localization to that of myoVb-tail expression. The transcytotic pathway moves from the basolateral early endosome to the common endosome before arriving at the ARE. Because cells were loaded with dIgA at the basolateral membrane, it is thought that the three peaks on the western blots may correlate with these three organelles, and the peak at fraction 25 may be the basolateral endosome. We reasoned that the more contaminating cellular structures that can be removed, the more accurate the proteomic analysis would be. The total protein concentration and lipid marker DiD show that the least contamination occurred within the 6–10 fraction peak of myosin Vb-tail expressing cells. Because fractions 6–10 were the least contaminated, and contained both myosin Vb-tail and dIgA, we decided to pool these fractions for further purification through fluorescence-activated sorting. (PDF) [file pone.0040826.s002.pdf]

Figure S2. Analysis of cellular fractions from iodixanol gradients to identify MIRE vesicles

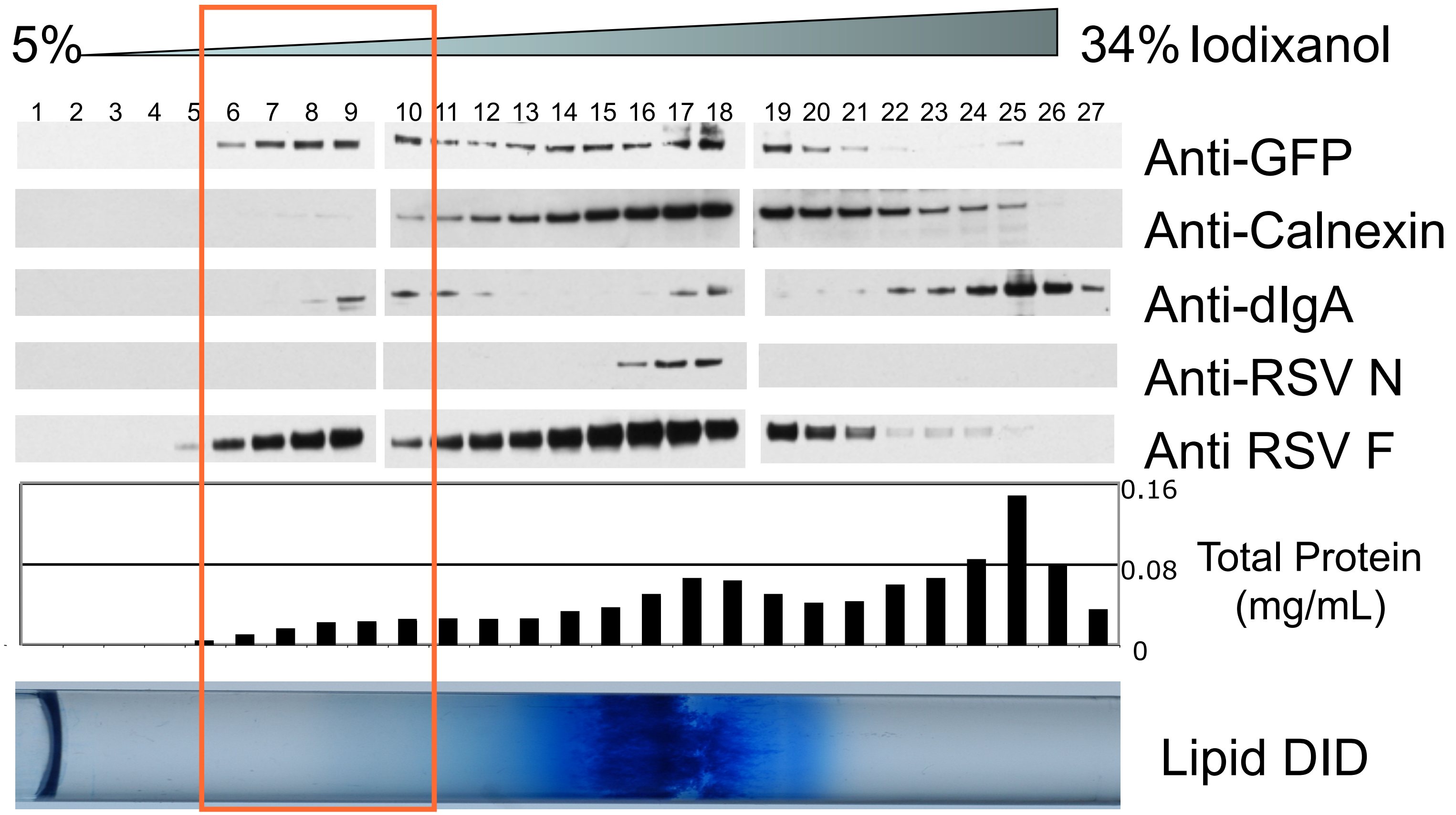

When the density gradient was analyzed for the myosin Vb-tail endosomes, we observed three peaks of myosin Vb localization. The first fraction of myosin Vb vesicles floated to the top of the gradient between fractions 6-10. The two additional peaks were located at fractions 18 and 25. Yet the latter two peaks were overlapping with the ER and other endosome components. When the density fractions were analyzed for the ER marker calnexin, the ER was found to localize across nearly the entire gradient peaking at fraction 18. A marker for the transcytotic pathway, dIgA, also showed three peaks. All three peaks are similar in localization to that of myoVb-tail expression. The transcytotic pathway moves from the basolateral early endosome to the common endosome before arriving at the ARE. Because cells were loaded with dIgA at the basolateral membrane, it is thought that the three peaks on the western blots may correlate with these three organelles, and the peak at fraction 25 may be the basolateral endosome. We reasoned that the more contaminating cellular structures that can be removed, the more accurate the proteomic analysis would be. The total protein concentration and lipid marker DiD show that the least contamination occurred within the 6-10 fraction peak of myosin Vb-tail expressing cells. Because fractions 6-10 were the least contaminated, and contained both myosin Vb-tail and dIgA, we decided to pool these fractions for further purification through fluorescence-activated sorting.
