# Supplementary material for: Respiratory Syncytial Virus Assembles into Structured Filamentous Virion Particles Independently of Host Cytoskeleton and Related Proteins
Source: PLoS One. 2012 Jul 13;7(7):e40826. doi: 10.1371/journal.pone.0040826 (PMC3396619; doi:10.1371/journal.pone.0040826)
Supplement: Figure S3 — Flow cytometric sorting for final purification of MIRE vesicles. Pooled gradient fractions were purified further through fluorescence-activated sorting. These pooled fractions were sorted through a series of gates based on the content of GFP, DiD and size to remove positive events that may be nonspecifically bound to another vesicle. The final sample contained 2% of the starting material, and showed greater than 90% specificity for GFP and DiD staining. When the pre- and post-sort material was analyzed by confocal microscopy, we observed little co-localization in the pre-sort material, whereas the post-sort material had high co-localization of both GFP and DiD. (PDF) [file pone.0040826.s003.pdf]

Figure S3. Flow cytometric sorting for final purification of MIRE vesicles

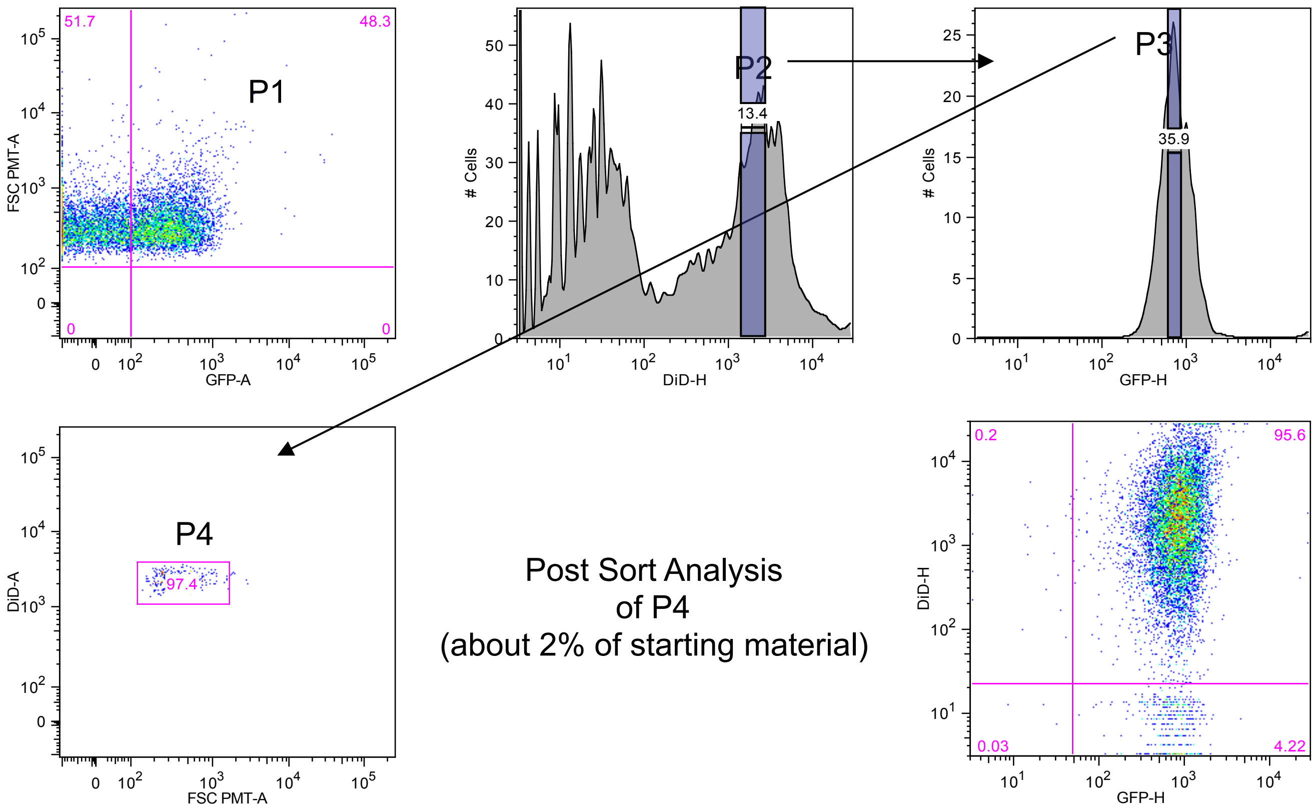

Pooled gradient fractions were purified further through fluorescence-activated sorting. These pooled fractions were sorted through a series of gates based on the content of GFP, DiD and size to remove positive events that may be nonspecifically bound to another vesicle. The final sample contained 2% of the starting material, and showed greater than 90% specificity for GFP and DiD staining. When the pre- and post-sort material was analyzed by confocal microscopy, we observed little co-localization in the pre-sort material, whereas the post-sort material had high co-localization of both GFP and DiD.
