# Supplementary material for: Respiratory Syncytial Virus Assembles into Structured Filamentous Virion Particles Independently of Host Cytoskeleton and Related Proteins
Source: PLoS One. 2012 Jul 13;7(7):e40826. doi: 10.1371/journal.pone.0040826 (PMC3396619; doi:10.1371/journal.pone.0040826)
Supplement: Figure S4 — MIRE vesicle proteomics list. The purified population of microsomes was reduced, denatured and subjected to SDS-PAGE. The sample was only electrophoresed about one cm into the gel, and Coomassie stained. This material that formed a smear then was excised, and proteins were in-gel digested and applied to a LC MS/MS mass spectrometer to generate tandem mass spectra and subsequently a list of proteins. High quality identification of proteins that occurred in two or more of five independent analyses (columns 1–5) are shown. Detection in the analysis is indicated by an X. (PDF) [file pone.0040826.s004.pdf]

Figure S4. MIRE vesicle proteomics list (1 of 2)

	Protein<br>**Cargo/membrane**	1	2	3	4	5
	α-1 catenin	X	X	X	X	X
	α-2 Integrin	X		X	X	X
	Annexin-A2	X	X	X	X	X
	α-V Integrin	X	X		X	X
	β-1 Integrin		X	X		X
	β-3 Integrin				X	X
	Basigin				X	X
★	β-catenin	X	X	X	X	X
	BSCv (Adipocyte plasma membrane-associated protein	X		X	X	
	CLIC 4	X			X	X
	δ-1 catenin	X	X	X	X	X
	EGFR kinase substrate 8	X			X	X
★	Endometrial Mucin-1	X				X
	Epithelial Cell Adhesion Molecule	X			X	X
	Galectin-3				X	X
	Galphai2	X	X			
	Guanine Nucletide B.P. Alpha 13 subunit	X			X	X
	Guanine Nucletide B.P. Alpha inhib				X	X
	Guanine Nucletide B.P. G(s) alpha subunit	X	X		X	
	Guanine Nucletide B.P. G(y) alpha subunit		X	X		
☆	MDR-1	X	X	X	X	X
	Na/K transporting ATPase	X	X	X	X	X
	PDZ domain containing protein1	X		X	X	X
☆	Plasma membrane Ca-transporting ATPase 1			X	X	
	Solute carrier family 2	X			X	
	Solute carrier family 3	X	X	X	X	
	Vacuole ATP synthase catalytic subunit	X	X	X	X	X

	Protein<br>**Vesicle Trafficking**	1	2	3	4	5
	Clathrin heavy chain	X		X	X	X
☆	EBP50	X	X	X	X	X
	IQGAP1	X		X	X	X
☆	Myosin 1b	X		X	X	X
	Myosin 1c	X		X	X	X
	Myosin 1d	X	X	X	X	X
	Rab10	X	X			X
★	Rab11	X	X			
	Rab7	X	X			X

	Protein<br>**Cytoskeletal**	1	2	3	4	5
	α-actinin1	X	X	X	X	X
	α-actinin2				X	X
	α-actinin4	X	X	X	X	X
☆	Actin	X	X	X	X	X
	Ankyrin-3	X				X
☆	Ezrin	X	X	X	X	X
	Keratin	X	X	X	X	X
☆	Moesin	X		X	X	X

	Protein<br>**Signalling**	1	2	3	4	5
	G protein-coupled receptor family C5				X	X
	Inhibitor of NFκB	X	X			
	Prostaglandin F2	X		X	X	X
	Src	X		X	X	X

★ Known ARE proteins
☆ Known Apical Proteins

The purified population of microsomes was reduced, denatured and subjected to SDS-PAGE. The sample was only electrophoresed about one cm into the gel, and Coomassie stained. This material that formed a smear then was excised, and proteins were in-gel digested and applied to a LC MS/MS mass spectrometer to generate tandem mass spectra and subsequently a list of proteins. High quality identification of proteins that occurred in two or more of five independent analyses (columns 1-5) are shown. Detection in the analysis is indicated by an X.

Figure S4. MIRE vesicle proteomics list (2 of 2)

**Protein**<br>**Unknown**	**1**	**2**	**3**	**4**	**5**
2,3-cyclic-nucleotide 3-phosphodiesterase (CNPase)			X	X	
Cytoplasmic FRM1 interacting protein 1	X			X	X
FARP1	X			X	X
FERM, RhoGEF		X	X	X	X
Ferritin L	X	X			X
GNB2				X	X
I-plastin	X		X	X	X
Junction Plakoglobin	X	X	X	X	X
KIAA1815	X		X		
Myoferlin	X	X			X
Niban	X	X			
Nicastrin	X	X			X
Plexin B2	X	X		X	X
PLS1	X				X
prolyl 4-hydroxylase	X			X	
Serine/Threonine-peotein kinase MST4	X			X	
Serine/Threonine-protein phosphatase PP1	X				X
Stomatin	X	X		X	X
thioredoxin	X		X	X	
Tissue- alpha-L-fucosidase	X	X			

**Protein**<br>**ER/Golgi**	**1**	**2**	**3**	**4**	**5**
Dolichyl-diphosphooligosaccaride	X		X	X	X
Endoplasmin	X	X	X	X	X
Gamaglutamyltranspeptidase1	X	X		X	X
Glutamyl aminopeptidase	X		X	X	
GRP78	X		X	X	X
HSP70	X				X
HSP71	X		X	X	X
N-acylsphingosine amidohydrolase	X	X			
Protein Disulfide isomerase A3	X		X	X	X
Protein Disulfide isomerase A4	X		X		X
Protein Disulfide isomerase A6	X				X

**Protein**<br>**Lysosomal**	**1**	**2**	**3**	**4**	**5**
Cathespin D	X	X		X	X
LAMP-1	X	X	X	X	

**Protein**<br>**Mitochondrial**	**1**	**2**	**3**	**4**	**5**
Aldehyde dehydrogenase 3B1	X	X	X	X	X
Cytochrome P450	X	X		X	X
Cytochrome P450 4A37	X		X	X	X

The purified population of microsomes was reduced, denatured and subjected to SDS-PAGE. The sample was only electrophoresed about one cm into the gel, and Coomassie stained. This material that formed a smear then was excised, and proteins were in-gel digested and applied to a LC MS/MS mass spectrometer to generate tandem mass spectra and subsequently a list of proteins. High quality identification of proteins that occurred in two or more of five independent analyses (columns 1-5) are shown. Detection in the analysis is indicated by an X.
