# Supplementary material for: Respiratory Syncytial Virus Assembles into Structured Filamentous Virion Particles Independently of Host Cytoskeleton and Related Proteins
Source: PLoS One. 2012 Jul 13;7(7):e40826. doi: 10.1371/journal.pone.0040826 (PMC3396619; doi:10.1371/journal.pone.0040826)
Supplement: Table S1 — Summary of candidate proteins from FCT Y2H screen of a custom lung cDNA library. A Y2H screen was performed by Hybrigenics (Paris, France) using the wt FCT peptide fused to LexA at the N-terminus as bait to screen a custom human lung cDNA library. The candidate proteins are listed by their gene name along with a description of the gene and associations. The cDNA fragment also was analyzed for being in frame (IF), unknown frame (??), and whether the interaction was with an antisense strand (antisense) or in the 3′ UTR (3′ UTR). (PDF) [file pone.0040826.s005.pdf]

Table S1. Summary of candidate proteins from FCT Y2H screen of a custom lung cDNA library

Gene Name	Frame	Y2H Fragments	Description
C1orf43	??	antisense	HepC NSSA Transactivated
COL6A2	IF		Collagen α2 (type IV) chain
DD5	IF		E3 ubiquitin protein ligase, HECT family
DGAT1	??	antisense	Diacylglycerol O-acyltransferase homolog 1
FAM114A2	??	antisense, 3′ UTR	Family with sequence similarity 114, member A2
FHL2	IF		Four and a half LIM domains 2: translational regulation
FLNA var1	IF		Filamin A; associated with actin cytoskeleton
GLUL	IF		Glutamate-ammonia ligase
GOLGA8A	??	antisense, 3′ UTR	Golgi autoantigen, golgi matrix protein
HLA-DRB5	IF		Major histocompatibility complex, class II, DRβ5
HPGD	??	antisense, 3′ UTR	Hydroxyprostaglandin dehydrogenase 15-(NAD)
HSPA5	IF		Heat shock 70kDa protein 5 (glucose-regulated protein, 78 kDa)
LPL	??	antisense, 3′ UTR	Lipoprotein lipase
RBM12	IF	3′ UTR, IF stop codon	RNA binding motif protein 12
RPL23A	??	antisense	Ribosomal protein L23a
SERPINF1	IF	IF stop codon	Serpin peptidase inhibitor, clade F
SGK269	??	antisense, 3′ UTR	Sugen kinase 269
SNRP70	IF		Small nuclear ribonucleoprotein 70kDa polypeptide (RNP antigen)
SP140	IF		SP140 nuclear body protein
SRP9	IF	antisense, 3′ UTR	Signal recognition particle 9kDa
TBC1D15	IF		TBC1 domain family, member 15; GTPase activating protein
TGFβ3	IF		Transforming growth factor β3

A Y2H screen was performed by Hybrigenics (Paris, France) using the *wt* FCT peptide fused to LexA at the N-terminus as bait to screen a custom human lung cDNA library. The candidate proteins are listed by their gene name along with a description of the gene and associations. The cDNA fragment also was analyzed for being in frame (IF), unknown frame (??), and whether the interaction was with an antisense strand (antisense) or in the 3´ UTR (3´ UTR).
