# Supplementary material for: Respiratory Syncytial Virus Assembles into Structured Filamentous Virion Particles Independently of Host Cytoskeleton and Related Proteins
Source: PLoS One. 2012 Jul 13;7(7):e40826. doi: 10.1371/journal.pone.0040826 (PMC3396619; doi:10.1371/journal.pone.0040826)
Supplement: Table S2 — Summary of results available antibodies and shRNAs. The available reagents for the FCT Y2H candidate genes are summarized. For genes with available antibodies, HEp-2 cells were inoculated with RSV wt strain A2 at an MOI = 1.0 for 24 hours. At 24 hours, cells were fixed, and RSV F and the indicated cellular protein were detected by indirect immunofluorescence. For genes with available shRNA lentivirus constructs, HEp-2 cells were transduced with each lentivirus construct. A heterogeneous population was selected using puromycin, and expression of the shRNA was confirmed by concomitant expression of GFP. Cells then were infected with RSV strain A2 at an MOI = 0.05 for 72 hours and both supernatant and cell associated virus yields were determined by plaque assay. The results for localization to viral filaments marked by RSV F and affect of shRNA expression on viral yields is summarized. (PDF) [file pone.0040826.s006.pdf]

Table S2. Summary of results available antibodies and shRNAs

Gene Name	Antibody	shRNA	Results
FHL2	+	+	No co-localization with RSV F; shRNA does affect on viral titers
FLNA, var 1	+	+	Co-localization with RSV F in viral filaments; shRNA does affect on viral titers
GLUL	+	+	No co-localization with RSV F; shRNA does affect on viral titers
SRP9	+	+	Antibody does not work for IF; shRNA does not affect viral titers
TBC1D15	+	+	Antibody does not work for IF; shRNA does not affect viral titers

The available reagents for the FCT Y2H candidate genes are summarized. For genes with available antibodies, HEp-2 cells were inoculated with RSV *wt* strain A2 at an MOI=1.0 for 24 hours. At 24 hours, cells were fixed, and RSV F and the indicated cellular protein were detected by indirect immunofluorescence. For genes with available shRNA lentivirus constructs, HEp-2 cells were transduced with each lentivirus construct. A heterogeneous population was selected using puromycin, and expression of the shRNA was confirmed by concomitant expression of GFP. Cells were then infected with RSV strain A2 at an MOI=0.05 for 72 hours and both supernatant and cell associated virus yields were determined by plaque assay. The results for localization to viral filaments marked by RSV F and affect of shRNA expression on viral yields is summarized.
